# Establishing a best practice for the freeze-thaw cycle of cryopreservation to approximate living muscle, joint, and associated soft tissue properties in cadaveric upper limbs

**DOI:** 10.1016/j.jpra.2025.08.012

**Published:** 2025-08-22

**Authors:** Cade R McGarvey, Summer M Drees, Austin Lawrence, Noah D Miller, Sahil Kapur, Vihan De Silva, Martin Skie, Ahmed Suparno Bahar Moni

**Affiliations:** a Department of Medicine, The university of Toledo, College of Medicine and Life sciences, 3000 Arlington Avenue, Toledo, OH 43614, USA; b Department of Orthopedics, The university of Toledo, College of Medicine and Life sciences, 3000 Arlington Avenue, Toledo, OH 43614, USA

**Keywords:** Cadaver, Upper limbs, Fresh-frozen, Freeze-thaw, Simulation, Surgical

## Abstract

**Introduction:**

Fresh-frozen cadavers are useful for surgical simulation and experimentation since they preserve many properties of live specimens, but achieving realistic physiological properties requires thawing that risks tissue damage.

**Methods:**

Ten fresh-frozen cadaveric upper limbs were frozen at −17 °C, refrigerated at 4 °C for 48 h, then thawed at room temperature for 8 h. The core temperature of the limbs was measured hourly. Joint flexibility, durometric hardness, and shore hardness were measured every two hours. Four limbs had muscle biopsies acquired every 2 h.

**Results:**

The limbs were above freezing after about 1 h of thawing. The upper limbs achieved a flexibility score of 3/5 after 2 h of thawing, mimicking healthy live limbs. The limbs became softer and more pliable with more time thawing. Muscle biopsies showed no tissue damage in any samples taken at h 2 and 4 of thawing. Three limbs showed no tissue damage at h 6 or 8, either. One limb had biopsies at 6 and 8 h demonstrating moderate to severe tissue damage.

**Conclusions:**

It was concluded that for ideal thawing, fresh-frozen cadaveric upper limbs should be thawed for 2 h after 48 h of refrigeration, permitting surgical simulation for 4 h after thawing, though potentially for 6 h.

## Introduction

Fresh-frozen cadavers have many uses in the medical field, including education and research.^1^, ^2^, ^3^ Fresh-frozen is often chosen over other preservation techniques because it allows for color, joint mobility, and tissue composition that is closest to reality.^1^^,^^4^ Studies have also found similarity in biomechanical properties of fresh-frozen cadavers and fresh specimens.^5^ Because of the value of fresh-frozen cadavers and their far-reaching utilization in many fields, it would be expected that a best practice method exists for preservation; however, the procedures used to freeze and thaw cadavers vary widely, and there is no universal agreement.^1^ However, procedures often have some common steps, like a period of freezing followed by one of thawing. The temperature at which cadavers are frozen varies from −17 °C to −40 °C.^1^ Some studies mention a thawing period, but do not specify the thawing temperature.^6^ Other procedures have thawed cadavers by refrigeration, at room temperature, or a combination of both.^7^, ^8^, ^9^ In a study analyzing the effect of repeated freeze-thaw cycles on human muscle tissue, Klop found that cadaver upper extremities had less of a change in CT attenuation from fresh tissue when thawed at 2 °C than when thawed at 19 °C.^7^ However, no analysis was done on thawing for some time at 2 °C, then at 19 °C. The purpose of this study is to investigate a best practice method for freezing and thawing cadavers that results in the closest emulation of a live specimen to improve the quality of fresh-frozen cadaver education and research.

## Methods

Autonomous consent free from coercion was obtained from the cadaveric donors or next of kin and was not secured from executed prisoners or prisoners of conscience. Two fresh-frozen cadaver upper extremities were frozen at −17 °C for at least 48 h. The upper extremities were placed in refrigeration at 4 °C, one for 24 h and the other for 48 h, before placement at room temperature. Once placed in room temperature, the limbs had their core temperature measured hourly, and their joint flexibility measured every 2 h until achieving a core temperature above 0 °C and a joint flexibility of 3.

The joint flexibility of both upper limbs in the preliminary trial was three at all points of measurement. The limb that 24 h of refrigeration had core temperatures of −2.2 °C, −2.2 °C, −1.6 °C, −1.3 °C, −1.3 °C, −0.3 °C, and 5.0 °C at h 0, 1, 2, 3, 4, 5, and 6, respectively. The limb that underwent 48 h of refrigeration had core temperatures of −1.1 °C, −0.9 °C, and 4.5 °C at h 0, 1, and 2, respectively.

Ten fresh, frozen cadaver upper extremities were frozen at −17 °C within 48 h post-mortem. The upper extremities were frozen for at least 48 h. Each upper extremity was then placed in refrigeration at 4 °C for 48 h. After the refrigeration period, the upper extremities were moved to room temperature.^9^ Immediately, a core temperature was measured by placing a thermometer in the proximal upper extremity. The temperature measurement was repeated at 1- intervals for the next 8 h.^10^ The upper extremities were also assessed for skin turgor, muscle tone, and joint flexibility at 2-h intervals for the next 8 h.^6^ Additionally, for four upper extremities, 3–4 mm tissue samples of the biceps brachii muscle were taken beginning at 2 h after the upper extremities were placed at room temperature. Biopsy samples were then repeated at 2-h intervals for the next 6 h. A total of four biopsy samples were collected from each of the four upper extremities. The samples were fixed in 10 % neutral buffered formalin and allowed to fix for 24–48 h before staining for histologic analysis utilizing hematoxylin and eosin (H&E). Each sample was evaluated under plane light with a binocular phase contrast microscope with 20x, 40x, and 100x magnification. Slides were assessed for tissue degradation and changes in muscle structure.

## Results

### Core temperature

The core temperature of the cadaveric upper limbs was measured hourly over 8 h immediately after the limbs were transferred to room temperature. The following average temperatures (*n*=10 cadaveric arms) were recorded (Table 1). The data indicates a consistent and gradual increase in core temperature over the 8 h. The initial low temperature of 0.3 °C at h 0 reflects the cadaver's state after being transferred from 4 °C storage. By h 8, the temperature had risen to 17.5 °C, approaching room temperature. This trend demonstrates the effectiveness of the thawing process that gradually brings the cadaveric tissue to a more stable thermal state.

**Table 1:** Hourly temperatures recorded for cadaveric upper limbs.

Limb	Initial	Hour 1	Hour 2	Hour 3	Hour 4	Hour 5	Hour 6	Hour 7	Hour 8
1	1.2	4.1	7.3	11.2	12.5	15.1	16.1	16.9	17.0
2	0.3	2.6	5.2	11.4	12.1	13.5	13.8	16.2	18.2
3	−0.7	1.5	6.6	10.4	14.5	16.3	18.7	19.4	20.1
4	−0.7	1.3	6.1	11.6	13.9	15.6	18.9	19.7	19.8
5	2.0	3.5	6.9	10.0	11.0	13.1	14.4	16.1	16.1
6	−0.4	0.6	3.5	6.2	7.9	10.6	12.9	14.5	15.8
7	2.7	4.3	6.6	10.0	10.5	12.3	14.3	16.1	17.5
8	−0.9	2.0	3.8	6.7	11.1	13.2	14.8	15.3	16.5
9	0.1	3.7	6.3	7.8	10.5	13.2	15.2	16.5	17.7
10	−1.0	−1.0	−0.5	3.8	4.9	7.8	13.0	15.1	16.5
Mean	0.3	2.3	5.2	8.9	10.9	13.0	15.2	16.6	17.5

Temperatures above freezing point ensure that the tissues are sufficiently thawed to allow for reliable manipulation and assessment without compromising structural integrity or biochemical properties. The temperature must be stable and uniform throughout the tissue to facilitate accurate and consistent results in further analyses such as histology, biomechanical testing, and other experimental procedures.

This indicates that the thawing procedure effectively brings the cadaveric tissues to a suitable temperature for subsequent experimental procedures, further confirming the protocol's reliability.

### Shore Hardness

#### Durometer Measurements

**Fig. 1:**
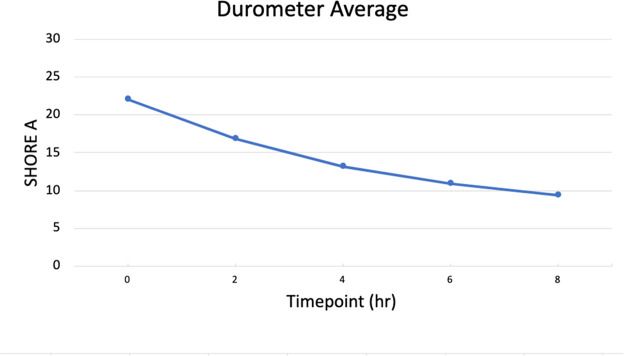
Average of ten cadaveric upper limbs Shore A Hardness from durometric measurements.

Skin turgor and muscle tone were assessed with a durometer using the Shore A hardness scale. Measurements were taken every 2 h over 8 h, starting immediately after the cadaveric arms were placed at room temperature. The following average Shore A hardness values were recorded from the 10 cadaveric arms (Fig. 1). The data indicate a progressive decrease in Shore A hardness values over the 8 h. Initially, at h 0, the high Shore hardness value of 22.1 reflects the rigidity of the skin and muscle tissue immediately after being brought to room temperature from a thawed state. As the h progressed, the Shore hardness values steadily decreased, reaching a value of 9.4 at h 8. This trend suggests that the skin and muscle tissue gradually became more pliable and less rigid over time, indicating successful thawing and partial restoration of normal tissue properties.

#### Manual measurements

**Fig. 2:**
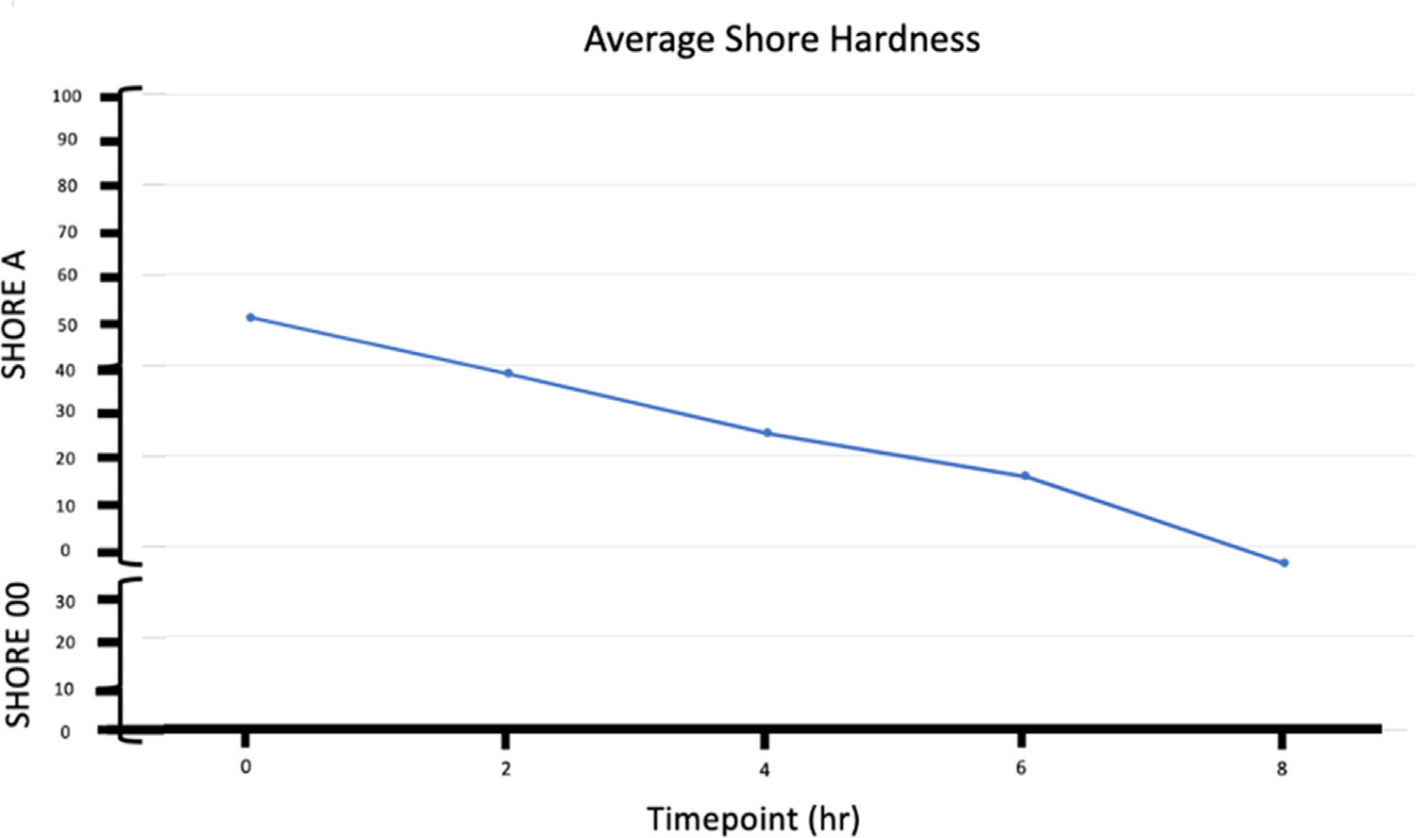
Average of ten cadaveric upper limbs Shore A and Shore 00 Hardness from manual measurements.

In addition to durometer measurements, manual measurements were taken using both Shore A and Shore 00 hardness scales (Fig. 2). The manual measurements show a similar trend to the durometer measurements, with a steady decrease in hardness values over the 8 h. Initially, the values were higher, reflecting greater rigidity, but they progressively decreased as the tissues thawed and softened. The transition to negative values by h eight indicates a significant reduction in hardness, corresponding to the softened state of the tissue.

#### Comparison of Durometer, Manual Shore Hardness Measurements, and Ideal Shore Hardness Range

As part of the literature review, a Shore hardness range for live human tissue, spanning from Shore 00 30 to Shore A 16, was identified. This range is suitable for accurately representing the biomechanical properties of soft tissues, ensuring reliable assessments for both clinical and research purposes. A Kruskal–Wallis test was performed to determine the similarity between the durometer measurements, manual Shore hardness measurements, and the ideal Shore hardness range (Shore A 16 to Shore 00 30) at each time point (Table 2) using a significance of *p* < 0.05.^11^^,^^12^ The comparison using the Kruskal-Wallis test shows that past 6 h, there was no statistical difference, demonstrating that the cadaveric tissue hardness measurements (both durometer and manual) are similar to the ideal Shore hardness range for human tissue. This suggests that the thawing protocol effectively restores tissue properties to a range suitable for subsequent experimental procedures. The results from both methods are consistent, demonstrating the reliability of the thawing protocol. However, reliance on subjective manual measurements for Shore Hardness should not be used alone, especially early in the thawing process.

**Table 2:** Kruskal-Wallis analysis of ideal human tissue hardness compared to durometric measurements and manual shore hardness measurements for ten cadaveric upper limbs’ muscle.

Time	Kruskal-Wallis Chi-squared score	Degrees of freedom	p-value
0 H	23.18	2	9.258 × 10^–6^ *
2 H	24.214	2	5.52 × 10^–6^ *
4 H	15.587	2	0.0004125*
6 H	8.9125	2	0.01161*
8 H	3.5822	2	0.1668

*p* < 0.05; *denotes statistically significant data.

#### Joint mobility

**Fig. 3:**
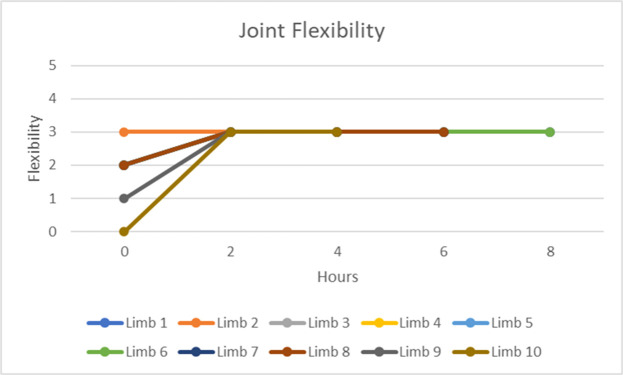
Joint mobility of ten cadaveric upper limbs.

To evaluate the joint mobility of the cadaveric arms, measurements were taken every 2 h over 8 h. Joint mobility was assessed using a scale from 0 to 6, where 0 indicates no mobility and 6 indicates complete instability. The ideal joint mobility value for cadaveric studies is 3. The following average values were recorded for the 10 cadaveric arms (Fig. 3). To determine how closely the joint mobility values approached the ideal value of 3, a Mann–Whitney test was performed for each time point (Table 3). At h 0, the joint mobility was significantly different than the ideal joint mobility of 3 (*p*<0.001). The joint mobility measurements show that the cadaveric arms achieved and maintained the ideal joint mobility value of 3 from 2 h to 8 h post-thawing. This reinforces the effectiveness of the thawing procedure in restoring joint mobility to a level suitable for cadaveric studies. The Mann-Whitney test further supports these findings, indicating no significant differences from the ideal value during these time points.

**Table 3:** Mann-Whitney analysis of average joint mobility of ten cadaveric upper limbs compared to the ideal value of 3.

Time	Mann-Whitney test result	p-value
0 H	*W* = 5	0.0001816 *
2 H	*W* = 50	NA
4 H	*W* = 50	NA
6 H	*W* = 40	NA
8 H	*W* = 20	NA

*p* < 0.05; *denotes statistically significant data.

#### Muscle Biopsy Degradation

**Fig. 4:**
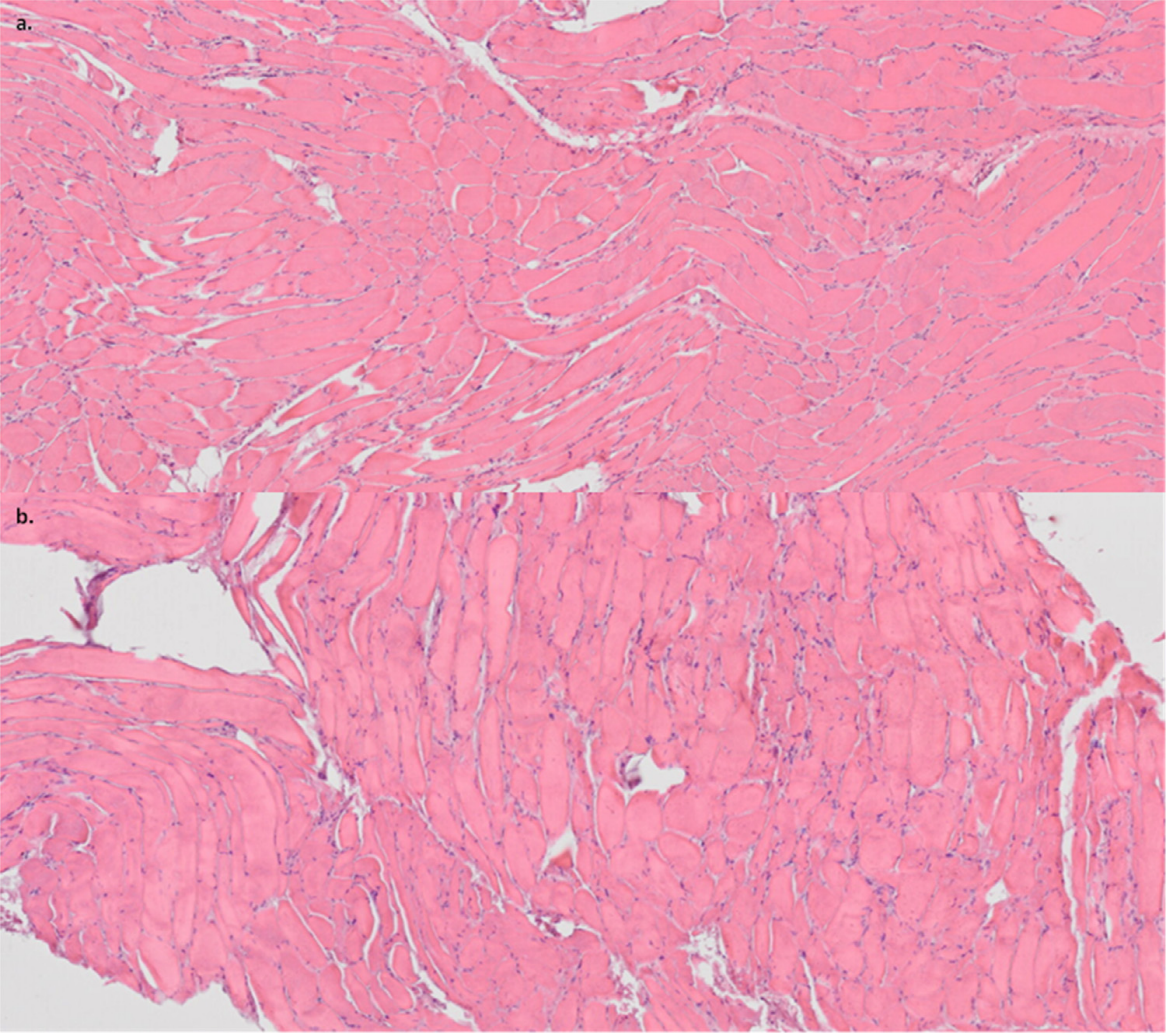
Limb 4 histology samples. a) Taken 2 h after placement at room temperature and was scored 0–1. b) Taken 8 h after placement at room temperature and was scored 2–3.

Muscle biopsies from 4 of the cadaveric upper limbs were taken at 2, 4, 6, and 8 h and prepared on slides for light microscopy with H&E staining. Analysis showed that on a scale of 0–1 indicating minimal to no damage, 1–2 indicating moderate damage, and 2–3 indicating severe damage, Limbs 1, 2, and 4 scored 0–1 at all time points and Limb 3 scored 0–1 at 2 and 4 h but scored 2–3 at h 6 and 8 (Fig. 4). To determine how closely the muscle degradation values approached the ideal scaled value of 0–1 (minimal to no damage), a Mann–Whitney test was performed at each time point. At 2 and 4 h, the Mann-Whitney test resulted in *W* = 8, with the p-values reported as “NA,” indicating that no significant differences were detected. At h 6 and 8, the Mann–Whitney test resulted in *W* = 6 with the p-values at 0.4533, indicating no significant deviation from the ideal range.

These findings suggest that muscle biopsy integrity did not significantly differ from the ideal range of 0–1, indicating that the freezing and thawing process causes minimal to no damage to cadaveric tissues. The structural integrity of the muscle is preserved, ensuring the tissues are suitable for research and educational purposes.

## Discussion

Cadavers have been useful for medical education and research. While they can simulate living tissue, there is a limit to their ability. Fresh-frozen cadavers preserve much of the pre-mortem structure of the tissues since the lack of preservative fluids prevents chemical reactions of the tissue, allowing the cadaver to be useful for surgical simulation. Working with fresh frozen cadavers does have the potential to cause harm. Cadavers can have infectious pathogens such as tuberculosis, viral hepatitis, AIDS, and prion diseases.^13^ The risk of disease transmission is higher when working with fresh-frozen cadavers as opposed to embalmed cadavers. However, embalming cadavers often significantly affects their color, joint mobility, and tissue properties, limiting their usefulness when a realistic tissue model is required.^1^^,^^4^^,^^5^ Certain safety measures can help prevent the transmission of pathogens. One important safety measure is to record the donor's cause of death and past medical history, if possible. Proper protective equipment should be worn. Proper decontamination and disinfection protocols should be followed.^13^ Even with safety measures carefully followed, there is still some risk of pathogen transmission, but following these practices can help lower the risk. Additionally, the risk of infection transmission is much lower in a cadaver than in a living person. Most pathogens have a short lifespan after the host dies.^14^

One example of a preservative fluid is formaldehyde, which stiffens and discolors the tissue.^15^ Another embalming method that produces preserved cadavers with greater similarity to live specimens than formaldehyde is the Thiel method. The Thiel method has been shown to preserve cadavers successfully without decreasing the flexibility of joints, stiffening the tissue, or altering the color of the anatomic structures.^16^ This, too, has antimicrobial properties, and the cadavers can last for years after undergoing the Thiel method. It has advantages over fresh-frozen cadavers in the greater amount of time specimens can be used before decay, the decreased risk of microbial growth, and decreased upfront costs.^17^ However, if upfront costs are already paid and proper facilities are in place, fresh-frozen cadavers are a simpler method than the Thiel method. Each comes with a risk of altering different biochemical properties.^1^^,^^17^ It was shown that the Thiel method could produce cadavers with better MR image quality than fresh-frozen cadavers or live patients. Although the image quality is better in Thiel, fresh-frozen cadavers modeled the live patient better in terms of image quality. It should be noted that the fresh-frozen cadavers were not thawed, which could affect results.^17^ The work described in this paper looked at the time it took for fresh-frozen human cadaveric upper limbs to thaw for optimal surgical simulation while minimizing tissue degradation.

Preliminary data for this study were used to inform the methodology regarding the time the upper limbs should spend in refrigeration. The limb that was in refrigeration for 24 h required 6 h of room temperature thawing to achieve a core temperature above freezing. The limb that was in refrigeration for 48 h required 2 h of room temperature thawing to achieve a core temperature above freezing. Prior work demonstrated that keeping the limbs at room temperature for longer periods causes significant tissue degradation, without the same observation for longer periods of refrigeration. This, in conjunction with our preliminary data, led us to incorporate a 48-h refrigeration period for our upper limbs and reject a 24-h refrigeration period.^7^

The core temperature shows a gradual increase over the 8 h. The overlap with the ideal range during these h suggests that the freeze-thaw process effectively stabilizes the tissue temperature, making it suitable for further handling and analysis without risk of thermal damage or enzymatic degradation. The durometer and manual Shore hardness measurements reveal a consistent decrease in hardness, with values aligning with the ideal range by h 6. This indicates that the thawing process successfully restores the tissue's natural pliability and elasticity, crucial for accurate biomechanical studies and realistic tissue manipulation in educational settings. Joint mobility values reaching the ideal score of 3 from 2 h onwards demonstrate that the freeze-thaw method effectively maintains the functional integrity of joints. This stability is essential for studies requiring realistic joint movement and flexibility, ensuring that experimental conditions closely mimic those of living tissue. Minimal to no degradation was observed in all muscle biopsies up to 4 h after placement in room temperature, and only 1 biopsy at 6- and 8-h showed degradation, confirming that the freeze-thaw process preserves cellular and structural integrity. The damage indicated the end of the ideal range for surgical simulation, which is why the authors terminated the experiment after 8 h of thawing at room temperature. Tissue integrity is critical for histological studies and ensures that the tissue remains viable for detailed microscopic analysis, maintaining its research and educational value.

This study demonstrates that there is a time window for upper limbs to be used. The window ends after approximately 6–8 h of room temperature thawing after 48 h of refrigeration. Although in some cases, it could be longer. For most limbs, this window allows for at least 4 h of potential operative time. Based on the data collected in this study, a recommended freeze-thaw procedure involves freezing cadaveric upper limbs at –17 °C for at least 48 h, thawing the limbs at 4 °C for 48 h, then thawing the limbs at room temperature for 2 h. At this point, the limbs should be ready for use and continue to be useful for approximately 4 h. The limbs could potentially last longer before significant tissue damage occurs. The limbs could likely require <2 h of thawing; however, flexibility measurements were taken every 2 h instead of hourly to minimize limb disturbance. This frequency of measurement could indicate that the time it takes to achieve a joint flexibility of 3 is less than what was measured. However, the scope of the recommended freeze-thaw procedure is limited to the use of human upper limbs separated from the rest of the body at the shoulder joint because this study did not include the rest of the cadaver. Additional work is necessary to detail freeze-thaw procedures for the rest of the body and whole cadavers.

The recommended procedure set forth by this study was modeled after previous studies; however, more detail was added to the methods so that this recommendation can be utilized in future studies as a freeze-thaw template to follow. Davenport, et al. used refrigeration at 4 °C for 24–48 h before thawing at room temperature for 4–8 h when working with upper limbs.9 Davenport, et al. is not unique in that most procedures in the literature for preparing fresh-frozen cadavers are not specific, and there is no standard preparation procedure.1 The change in attenuation of cadavers thawed at 2 °C was less than those thawed at 19 °C, indicating less limb decomposition with thawing at 2 °C than 19 °C.^7^ To minimize decomposition of the upper limbs used, the time spent thawing at 4 °C was set at 48 h. This, in turn, decreased the amount of time necessary for thawing at room temperature. Although this required a longer overall thawing process, these studies cited indicate that doing so allowed more time for the cadaveric upper limbs to be useful for surgical simulation before tissue decomposition affected the quality of the limbs. With proper planning in most educational, training, and experimental settings, the additional refrigeration time can decrease the odds of room temperature thawing to the desired temperature later than anticipated or needed. This, in turn, can prevent the need for restorage and later use, requiring another freeze-thaw cycle and increasing tissue degradation.^7^ Variation in exact time needed for thawing depends on the size of the cadaveric specimen, so the core temperature of the limb should be kept in mind as the end goal rather than the time spent thawing the specimen. The recommended procedure for freeze-thaw, as detailed in this study, is based on its effectiveness in achieving the desired temperature and the presence of minimal tissue degradation that occurs in the same time frame.

This study had strength from using clinically relevant data, like joint mobility and tissue structural integrity, to compare with temperature and time data. Clinically relevant data provided a better understanding of when the upper limbs most realistically mimicked live specimens. Limitations of this study included the frequency of data collection. To minimize disturbance of the specimens, joint mobility, skin turgor, durometric, and biopsy data collections were taken every 2 h instead of hourly, as was done with temperature recordings. More frequent measurements could provide a more precise time window where the upper limbs can be used. It is also recommended for future work that the time of data collection be extended to greater than 8 h after commencing room temperature thawing. Although tissue degradation was observed by h 6, it only occurred in one limb and was not statistically significant. Extension of data collection until a statistically significant number of muscle biopsies have experienced tissue destruction could also enhance the understanding of how long upper limbs can be used for surgical simulations after thawing. The refrigeration methods from this study were based on the work by Davenport, et al. and Klop, et al. however, refrigeration for 48 h without room temperature thawing was in some cases sufficient for achieving a core temperature above freezing.^7^^,^^9^ This demonstrates the possibility that longer periods of refrigeration could allow for thawing upper limbs to the point that they become capable of use for surgical simulation earlier in room temperature thawing, while still delaying tissue decomposition.

## Conclusion

The results across all tests confirm that the freeze-thaw protocol effectively preserves the integrity, functionality, and suitability of cadaveric tissues during the 6-h time range after the tissue has been placed at room temperature. The method ensures that tissues quickly achieve and then maintain ideal properties, supporting high-quality and reliable experimental outcomes for medical research and educational endeavors. It is recommended that cadaveric upper limbs frozen at −17 ⁰C thaw via 48 h of refrigeration at 4 ⁰C, then be placed at room temperature for 2 h before use in surgical simulation. The upper limb could be used for approximately 4 h before the structural integrity of the tissue becomes compromised.

## CRediT authorship contribution statement

**Cade R McGarvey:** Conceptualization, Writing – original draft, Writing – review & editing, Visualization, Methodology, Formal analysis, Project administration, Investigation. **Summer M Drees:** Conceptualization, Investigation, Methodology, Formal analysis, Writing – review & editing, Writing – original draft, Visualization, Project administration. **Austin Lawrence:** Investigation, Methodology, Writing – original draft, Writing – review & editing. **Noah D Miller:** Investigation, Methodology, Writing – original draft, Writing – review & editing. **Sahil Kapur:** Investigation, Methodology, Writing – original draft, Writing – review & editing. **Vihan De Silva:** Methodology, Investigation, Writing – review & editing. **Martin Skie:** Conceptualization, Resources, Writing – review & editing, Funding acquisition. **Ahmed Suparno Bahar Moni:** Conceptualization, Investigation, Methodology, Writing – review & editing, Resources, Supervision, Funding acquisition, Formal analysis.

## Declaration of competing interest

The authors report no conflicts of interest and no financial disclosures.
